# An Unusual Cause of Epistaxis: Paranasal Sinus Myeloid Sarcoma

**DOI:** 10.1155/2019/1312630

**Published:** 2019-02-12

**Authors:** Karuna Dewan, John H. Baird, Courtney B. Shires

**Affiliations:** ^1^Department of Otolaryngology Head and Neck Surgery, Stanford University, Stanford, USA; ^2^Division of Hematology, Department of Medicine, Stanford University, Stanford, USA; ^3^Department of Otolaryngology Head and Neck Surgery, The University of Tennessee Health Sciences Center, Memphis, USA

## Abstract

We report a case of a 65-year-old female who presented with right-sided headaches, blurring of vision in the right eye, cold-induced epistaxis, and facial numbness in the trigeminal nerve distribution. Laboratory studies revealed a significant number of myeloblasts on peripheral smear with granulated cytoplasm, irregular nuclei, and prominent vacuoles. Magnetic resonance imaging (MRI) of the brain demonstrated a T1-enhancing 1.5 cm right-sided dural-based lesion involving the medial sphenoid wing, cavernous sinus, infratemporal fossa, and sphenoid sinus region. An endoscopic biopsy of the lesion within the sphenoid sinus confirmed the diagnosis of myeloid sarcoma, with myeloblasts comprising 30% of cellularity by flow cytometry. A subsequent bone marrow biopsy revealed a hypercellular marrow with 23% blasts by flow cytometry that demonstrated a similar immunophenotypic pattern to those seen in the sinus mass. Fluorescence in situ hybridization (FISH) testing revealed the balanced translocation t(8;21)(q22;q22.1), consistent with a diagnosis of acute myeloid leukemia with *RUNX1-RUNX1T1*-balanced translocation by WHO 2016 criteria. Myeloid sarcoma represents a rare extramedullary presentation of acute myeloid leukemia (AML), either alone or in conjunction with blood or bone marrow involvement. This case emphasizes the need for a broad differential diagnosis and an aggressive work-up for any unusual paranasal sinus mass.

## 1. Introduction

Myeloid sarcoma (also known as granulocytic sarcoma or chloroma) is a rare extramedullary clinical presentation of acute myeloid leukemia (AML) seen in less than 10% of patients [1], in which a discrete mass of myeloblasts infiltrate and efface normal tissue architecture. Designated as a separate entity in the WHO 2016 classification [2], this extramedullary disease often presents concurrently or subsequent to systemic or bone marrow involvement with any subtype of AML, but in rare instances, can occur in isolation. These tumors may be found in any location including bone, periosteum, soft tissues, and lymph nodes, and less commonly the orbit, intestine, mediastinum, epidural region, uterus, and ovary [3–5]. Myeloid sarcoma is most often associated with AML with a monocytic differentiation (French-American-British (FAB) M4 or M5 classifications) or with AML harboring the cytogenetic abnormality t(8;21) [5] (also known as core binding factor (CBF) AML). With greater understanding of genetic and molecular risk factors, the prognosis of AML is now represented across a diverse spectrum, with favorable risk subtypes such as CBF AML achieving long term survival with intensive chemotherapy alone, and adverse risk subtypes often requiring more intensive therapy with allogeneic stem cell transplant [6–8].

We describe here a 65-year-old woman presenting with headache and cold-induced epistaxis. On subsequent work-up, she was found to have a sphenoid sinus myeloid sarcoma as the presenting sign of underlying AML.

## 2. Case Presentation

**Figure 1:**
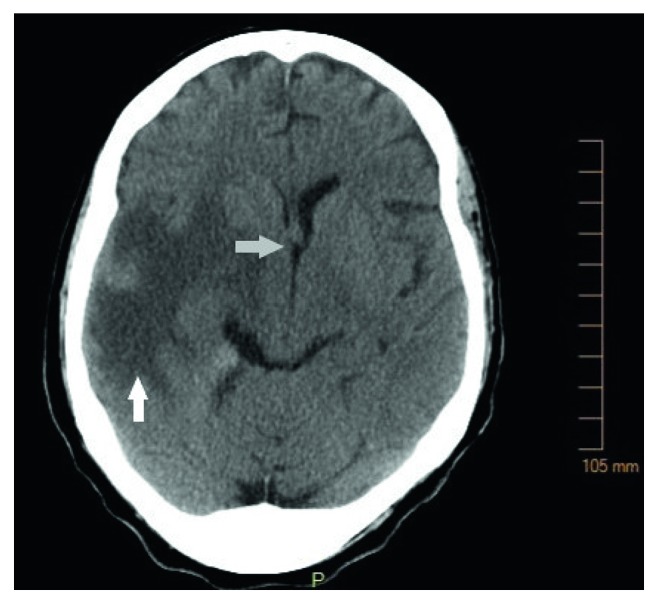
Computed tomography (CT) head imaging demonstrating a large focus of edema involving the right temporal lobe (white arrow) as well as hyperdense dural thickening in the right medial petrous apex with 6 mm of midline shift (grey arrow).

**Figure 2:**
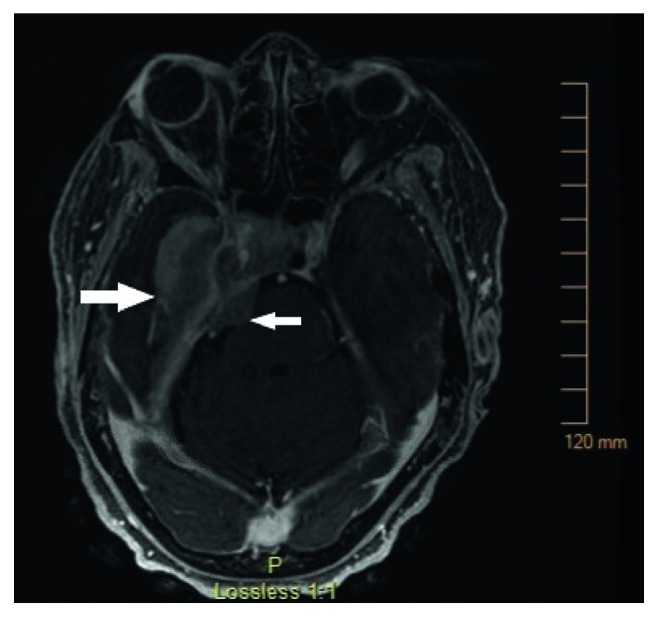
T1-weighted magnetic resonance imaging (MRI) of the head demonstrating abnormal signal along the dural surface of the right medial petrous region (smaller arrow) and paraclinoid region as well as sphenoid bone and cavernous sinus with upper sphenoid tumor extension (larger arrow), also extending to the foramen ovale and into the upper margins of the right infratemporal fossa.

A 65-year-old female presented to the emergency room with a complaint several weeks of worsening of right-sided headache, nausea, vomiting, cold-induced epistaxis, and blurring of vision from the right eye. Over the previous two months, the patient developed right facial numbness, 25-pound weight loss, and increasing right tongue swelling. On physical examination, the patient was noted to have disconjugate gaze to the right, as well as a right cranial nerve VII and XII palsy. Laboratory studies reveled 10% circulating blasts; there was no evidence of coagulopathy or tumor lysis syndrome. On review of the peripheral blood smear, there was a mixture of myeloblasts and promonocytes with granulated cytoplasm, irregular nuclei, and prominent vacuoles. Computed tomography (CT) imaging of the head performed in the emergency department demonstrated a large focus of edema involving the right temporal lobe as well as hyperdense dural thickening in the right medial petrous apex with 6 mm of midline shift (Figure 1). Magnetic resonance imaging (MRI) [9] demonstrated abnormal T1-intense signal along the dural surface of the right medial petrous region and paraclinoid region as well as sphenoid bone and cavernous sinus with upper sphenoid tumor extension, also extending to the foramen ovale and into the upper margins of the right infratemporal fossa (Figure 2). Imaging performed 2 months prior as part of surgical planning for a right mastoidectomy for chronic mastoiditis showed no evidence of this mass.

The patient was taken to the operating room for endoscopic biopsy of tissue in the sphenoid sinus, which subsequently demonstrated a myeloperoxidase- (MPO-) positive blast population infiltrating the sinus mucosal and bony tissue, positive for CD34, CD117, HLA-DR, CD33, CD11b, and partial CD13 by flow cytometry, consistent with a myeloid sarcoma. Positron emission tomography-computed tomography (PET-CT) was performed that showed intense fluorodeoxyglucose (FDG) avidity in the right temporal lobe, paraclinoid region, sphenoid bone, cavernous sinus, and infratemporal fossa on the right. A subsequent bone marrow biopsy demonstrated a hypercellular marrow with 23% myeloblasts with similar morphologic and immunophenotypic characteristics to the sinus myeloid sarcoma tissue. FISH analysis showed a t(8;21)(q22;q22.1) in all myeloblasts on metaphase analysis, without any additional cytogenetic abnormalities, consistent with a diagnosis of acute myeloid leukemia (AML) with a balanced translocation of *RUNX1-RUNX1T1*. Subsequent PCR-based testing demonstrated no mutations in *c-KIT*.

The patient was transferred to the inpatient hematology service and initiated induction chemotherapy with cytarabine and daunorubicin (7 + 3 regimen). Her induction course was complicated by hyperbilirubinemia, likely drug-induced. A repeat bone marrow biopsy obtained on day 12 demonstrated no increase in blasts but residual positivity for t(8;21) by FISH in 9 out of 20 metaphases; however, this resolved on repeat biopsy 1 week later. Repeat MRI showed residual tumor involving the right Meckel cave and cavernous sinus. Because of this, she went on to receive consolidative radiotherapy involving the whole brain, with follow-up MRI confirming complete resolution of the mass.

Given the patient's favorable risk disease, she proceeded to consolidative chemotherapy with high-dose cytarabine (HiDAC) alone. After 1 cycle, she presented with increasing fatigue and generalized weakness, and repeat bone marrow biopsy confirmed a relapse of AML, with 23% blasts negative for any evidence of the prior t(8;21), but demonstrating a new isolated del(20)(q11.2) in 9 out of 20 metaphases on karyotypic analysis. Lumbar puncture was performed and was negative for any leukemic involvement. She received salvage chemotherapy with G-CLAM, with her subsequent course complicated by neutropenic enterocolitis and recurrent drug-induced hyperbilirubinemia. She experienced delayed count recovery, with a repeat bone marrow biopsy demonstrating no residual disease. She was started on growth factor support and discharged home, where 1 week later, she experienced altered mental status and a fall. Her initial CT scan demonstrated left frontal trauma with associated intraparenchymal hemorrhage in the left frontal and right temporal lobes in the setting of thrombocytopenia. After a slow and incomplete recovery in the neurologic critical care unit, the family elected to take the patient home on hospice care, and she subsequently passed away.

## 3. Discussion

To our knowledge, there are fewer than 20 reported cases of nasopharyngeal myeloid sarcoma reported in the literature (Table 1), with a majority occurring concurrently with systemic disease and having contiguous orbital involvement [3, 10]. None of the previously reported cases describe disease contained entirely within the paranasal sinuses as in the current case.

Myeloid sarcoma has an association with several cytogenetic abnormalities, most commonly *MLL* rearrangements and t(8;21) and to a lesser degree monosomy 7, trisomy 8, inv(16), trisomy 4, monosomy 16, del(16q), del(5q), del(20q), and trisomy 11 [11–13]. Although myeloid sarcoma may occur at any age, it is more common in younger patients likely owing to the prevalence of *MLL* rearrangements and splitting. Approximately 16% of myeloid sarcoma cases carry concurrent NPM mutations, which are often associated with normal cytogenetics and FAB M4 or M5 morphologic classification. The accurate diagnosis of these cases can be challenging, particularly when there is no evidence of systemic disease, and imaging features are not sufficiently specific to distinguish myeloid neoplasm from other tumors including lymphoid and nonhematopoietic tumors.

PET-CT scan can be useful in the diagnosis and monitoring of disease progression in myeloid sarcoma. Cribe et al. noted PET-CT resolution of disease in organs correlated with resolution of bone marrow disease [14]. While two other author groups, Stozel et al. and Ravulapati et al., have noted that bone marrow can appear cleared of disease, PET-CT scan demonstrates remaining organ disease [15, 16]. In the case presented here, PET-CT scan was done initially for staging purposes and was not repeated.

There is a lack of definitive data regarding what prognostic significance extramedullary disease has in the context of concurrent systemic disease, if any. Early retrospective cohort studies suggested that the presence of concurrent extramedullary disease conferred an adverse impact; in one larger study, the 2-year event-free survival rate and overall survival rate was 32% and 43% versus 18% and 29% in isolated myeloid sarcoma and in concurrent systemic disease, respectively [10]. However, similar cohorts have also shown that, in isolated myeloid sarcoma, AML will almost inevitably arise with a median time to development of 5–12 months from diagnosis. Thus, the current consensus is that while patients with myeloid sarcoma have an overall poor prognosis with 5-year survival rates between 20 and 30% [4, 17–19], this appears only mildly and perhaps nonsignificantly worsened compared to age- and risk-matched survival in AML without extramedullary disease.

Given the high prevalence of concurrent systemic disease, most patients with myeloid sarcoma require conventional intensive induction chemotherapy combining an anthracycline and cytarabine at the time of diagnosis. Complete response rates to this conventional AML-type treatment vary significantly, from 94.4% to 67.4% [20] in favorable and adverse risk groups, respectively. The impact of extramedullary disease on treatment response and survival in favorable risk AML, in particular, those with t(8;21) treated with chemotherapy alone, remains unclear after several conflicting studies [21–23]. For patients who experience an incomplete response to this initial therapy or in those patients who have isolated myeloid sarcoma, some studies have shown excellent locoregional control with the use of consolidative radiotherapy [24, 25] though it remains unclear whether this intervention has an impact on overall survival.

In those patients who achieve a complete remission with their initial chemotherapy regimen, the uncertain prognostic implications of extramedullary disease in conjunction with the lack of randomized controlled studies comparing the optimal consolidative protocol often result in the use of allogeneic hematopoietic stem cell transplant (HCT) for eligible patients. The largest case series utilizing this approach in patients with myeloid sarcoma showed 5-year overall survival of 47% [26]. With the advent of routine testing for minimal residual disease (MRD), either through multiparametric flow cytometry or through PCR monitoring of targetable fusion transcripts (e.g., RUNX1-RUNX1T1 in AML with t(8;21)(q22;q22.1)), these results will likely be improved for selected patients with MRD-negative complete remissions.

While these tumors are quite rare, they can have unusual presentations in the head and neck region that result in symptoms such as exophthalmos, facial paralysis, or adenopathy [27]. It is important for the head and neck surgeon to consider this diagnosis as part of a wide differential so that a complete evaluation to evaluate for systemic disease can be undertaken. While the prognosis for myeloid sarcoma remains guarded, with the advent of more comprehensive genetic and molecular risk profiling and targeted treatment intensification based on these results, the results of early and aggressive treatment are likely to yield improved long-term outcomes.

## Figures and Tables

**Table 1:** Known cases of head and neck myeloid sarcoma reported within the literature. There are no reported cases with disease confined to the paranasal sinuses [10].

Case no.	Age (yr)/gender	Myeloid sarcoma site	Presentation	Bone marrow disease
1	77/F	Tongue	Tongue lesion	Yes
2	55/M	Mandible	Jaw pain	Yes
3	47/F	Tonsils, base of tongue	Sore throat	Yes
4	6/1M	Tonsil	Throat pain	Yes
5	25/M	Eye, parotid and submandibular gland	Bulging of right eye	Yes
6	79/M	Cutaneous cheek and eyelid lesion	Skin lesions	Yes
7	65/F	Lip	Lip mass	Yes
8	63/M	Cutaneous cheek lesion	Pimple	Yes
9	80/M	Cutaneous eyelid, forehead lip lesion	Skin rash	No
10	55/M	Gingiva, maxilla	Gingival mass	Yes
11	21/M	Tonsil	Enlarged tonsil	Yes
12	48/M	Mandible, maxilla	Unknown	Yes
13	77/M	Maxilla, alveolus	Mouth pain, dyspnea, fatigue	Yes
14	56/M	Mandible	Blasts in blood	Yes
15	85/F	Lip	Lip and skin lesion	Yes
16	69/M	Middle turbinate (intranasal)	Sinus pain and pressure	Yes
17	17/M	Cutaneous cheek lesions, breast mass	Skin lesion and breast mass	No
